# NCAPH plays important roles in human colon cancer

**DOI:** 10.1038/cddis.2017.88

**Published:** 2017-03-16

**Authors:** Liang Yin, Li-Ping Jiang, Qiu-Shuo Shen, Qiu-Xia Xiong, Xiao Zhuo, Long-Long Zhang, Hai-Jing Yu, Xiang Guo, Ying Luo, Jian Dong, Qing-Peng Kong, Cui-Ping Yang, Yong-Bin Chen

**Affiliations:** 1 The First Affiliated Hospital of Kunming Medical University, Kunming 650223, China; 2 The Third Affiliated Hospital of Kunming Medical University, Kunming 650223, China; 3 Key Laboratory of Animal Models and Human Disease Mechanisms, Kunming Institute of Zoology, Chinese Academy of Sciences, Jiao Chang Dong Lu 32, Kunming 650223, China; 4 Kunming College of Life Science, University of Chinese Academy of Sciences, Beijing 100049, China; 5 College of Life Sciences, Yunnan University, Kunming 650091, China; 6 Faculty of Medicine, Kunming University of Science & Technology, Chenggong County, Kunming 650500, China; 7 State Key Laboratory of Genetic Resources and Evolution, Kunming Institute of Zoology, Chinese Academy of Sciences, Kunming 650223, China

## Abstract

Colon cancer (CC) is one of the major malignancies worldwide, whose pathogenesis is complex and requires the accumulated alteration of multiple genes and signaling pathways. Condensins are multi-protein complexes that play pivotal roles in chromosome assembly and segregation during mitosis, meiosis and even tumorigenesis. Using tissue microarrays by immunohistochemistry and hematoxylin–eosin staining, we found that non-SMC condensin I complex subunit H (NCAPH) in colon cancerous tissues was higher than that in all corresponding adjacent non-cancerous tissues. We then characterized the exact function of the NCAPH in CC. We provided evidences showing that NCAPH is highly expressed in colorectal cancer cell lines comparing with normal human colonic epithelial cells, and identified many NCAPH mutations in CC patients. We found that depletion of NCAPH inhibits CC cell proliferation, migration *in vitro* and xenograft tumor formation *in vivo*. Furthermore, NCAPH knockdown promotes cell apoptosis and cell cycle arrest at G2/M phase. Interestingly, the NCAPH high expression in tumor tissues of colon patients had a significantly better prognosis and survival rate than low-expression patients, suggesting that NCAPH high expression promotes colonic cancerous cell proliferation; on the other hand, it may also sensitize these cells responding to chemo- or radio-therapies. Collectively, these findings reveal an important role of NCAPH in CC, indicating that NCAPH could be used as a new therapeutic target in future.

Colon cancer (CC) is one of the most common malignancies and the leading cause of cancer-related mortality among both men and women worldwide, which accounts for roughly 1.2 million new cases and 600 000 deaths per year.^1, 2, 3, 4^ The highest incidence has been reported in countries of Europe and North America, however, rapid increases in low-risk countries, such as in eastern Europe and east Asia, have recently been documented, which resulted from changes in dietary patterns and risk factors towards a so-called western lifestyle.^4, 5^ The prognosis of patients with CC has been improved during the past decades in many countries, its invasion and metastasis is one of the biggest problems in the clinical treatment, and is the most common reason for treatment failure. The complicated mechanisms include invasion of tumor cells, extracellular matrix degradation, epithelial–mesenchymal transition and angiogenesis of the tumor microenvironment. Lymph node metastasis is the main transfer way, which decides CC staging, auxiliary treatment plan and prognosis evaluation. In addition, accumulated alteration of multiple genes and signaling pathways is also one of the major reasons.

Condensins are multi-protein complexes that play a central role in chromosome assembly and segregation during mitosis and meiosis.^6, 7^ Many eukaryotic cells possess two different types of condensin complexes, known as condensin I and condensin II, each of which is composed of sets of subunits.^8, 9^ Condensins I and II share the same pair of core subunits, CAP-E/SMC2 and CAP-C/SMC4, both belonging to a large family of chromosomal ATPases, the SMC (structural maintenance of chromosomes) family whose members are involved in many aspects of higher-order chromosome dynamic, and three different non-SMC subunits.^10, 11, 12^ The canonical condensin complex (henceforth condensin I) plays an essential role in mitotic chromosome assembly and segregation from yeast to humans.

Non-SMC condensin I complex subunit H (NCAPH) is one of the three non-SMC subunits in condensin I, which belongs to a recently defined superfamily of proteins termed kleisins.^13^ Another two non-SMC subunits, CAP-D2 and CAP-G, share a highly degenerate repeating motif known as HEAT repeat.^14^ Some studies show that each subunit is essential for viability and plays an important role in mitotic chromosome architecture and segregation.^15, 16, 17^ To date, there has been no relevant report about the functional role of NCAPH in CC.

Prognostic biomarkers are key to the treatment of patients with CC and the decision to recommend adjuvant chemotherapy in patients with early-stage disease.^18^ At present, tumor grade, TNM stage and patient gender remain the most important among a handful of prognostic variables that are considered in the development of algorithms for the treatment of patients with early-stage CC.^19, 20^ In this study, we analyzed the relationship between NCAPH expression, and the pathogenesis and prognosis of CC. We found that NCAPH protein expression in CC tissues was higher than that in corresponding adjacent non-cancerous normal tissues; in addition, the NCAPH high expression in tumor tissues of CC patients had a significantly better survival rate than NCAPH low-expression patients. We also found that knockdown of NCAPH inhibits CC cells proliferation, migration *in vitro* and xenograft tumor formation *in vivo*, possibly through inhibiting cell cycle transition and inducing cellular apoptosis. Our findings indicate a critical role of *NCAPH* gene in the cure and prognosis of CC, which provide a possible new direction for CC research.

## Results

### NCAPH expression in colon tumors

**Figure 1:**
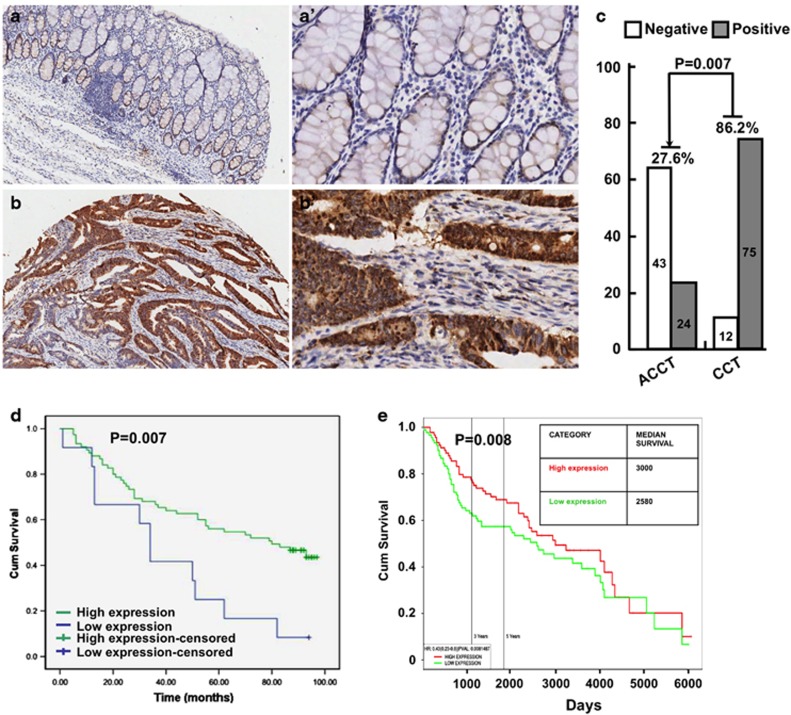
NCAPH expression in colon cancers. (**a–b′**) Colon cancer tissue microarray (TMA) that contained 90 colon cancer and paired adjacent normal or non-cancerous tissues were stained with NCAPH antibodies. Representative images with differential magnification (× 10 and × 40) of immunohistochemistry (IHC) staining were shown. (**a–a′**) NCAPH expression is low in normal tissues. (**b**–**b**′) NCAPH expression is high in colon cancerous tissues. (**C**) Quantification data for IHC. *P*=0.007. ACCT, adjacent colon cancer tissues (non-cancerous normal colon tissues); CCT, colon cancer tissues. (**d** and **e**) Kaplan–Meier plotter was used to analyze the protein expression data from IHC (**d**) and web resource transcriptional RNA data (**e**). NCAPH high expression in colon cancers had a significantly better survival rate than low-expression patients. Different color lines correlated with different NCAPH protein (**d**: *P*=0.007) or RNA (**e**: *P*=0.008) expression levels

Condensins are multi-protein complexes that play pivotal roles during mitosis, meiosis and tumorigenesis, NCAPH is one of the three non-SMC subunits in condensin I. Here we investigated the correlation between the expression of NCAPH protein and clinicopathological features of CC, and applied immunohistochemistry in the CC tissue microarray (TMA; Figures 1a–c), which contained 90 CC and paired adjacent normal or non-cancerous tissues (hereafter referred to as normal tissues). Ninety colorectal carcinoma cases were assessed from July 2006 to August 2014, including 47 males and 42 females, median age of patients was 69.71±11.06 years old, ranging from 48 to 90 years old. All specimens were graded using the pathologic and clinic stage, 48 cases were in stage I, II and 42 cases in stage III. According to TNM staging, 8 cases were in stage 1, 48 cases in stage 2, 30 in stage 3 and 2 cases were in stage 4. All tumors were adenocarcinomas, and the cases with the tumor size >5 cm (contain 5 cm) is 45, whereas <5 cm is 44 (Supplementary Table 1).

It has been demonstrated that condensing I complex is excluded from nucleus until nuclear envelope breakdown, which is in line with the findings that no inhibition of prophase chromosome condensation occurs after condensing I knockdown.^21, 22^ Consistent with the spatial regulation of NCAPH, both cytoplasm and nuclear expressions of NCAPH were detected in colon tumors and adjacent normal tissues, and NCAPH expression was higher in colon cancerous tissues than that in the normal tissues (Figures 1a–b′). The percentage of positive intensive NCAPH expression is significantly higher in colon cancerous tissues (86.2% 75/87) than that in normal colon tissues (27.6% 24/87), respectively (Figure 1c and Supplementary Tables 2 and 3). Interestingly, we analyzed the association of NCAPH protein expression with overall survival, and found that NCAPH high-expression patients had a significantly better survival rate, whereas in adjacent normal tissues had no significant prognosis and survival rate differences, comparing with NCAPH low-expression patients (Figure 1d and Supplementary Table 3). In addition, by applying web resource (PROGgeneV2, Indiana University) containing the transcriptional RNA dataset for colon cancer patients (GSE41258 and Saffer *et al.*^23^), the median survival time for patients with high expression of NCAPH was 3000 days, comparing with 2580 days for patients with low NCAPH expression (Figure 1e). We hypothesized that as one of the three non-SMC subunits in condensin I, NCAPH plays an important role in chromosome assembly and segregation during mitosis and meiosis, high expression of NCAPH might be important for colon cancerous cell proliferation; on the other hand, it might also sensitize the colon cancerous cells responding to chemo- and/or radio-therapies, which in turn results in better survival rate.

### NCAPH is highly expressed in colonic cancerous cell lines and mutated in colon cancers

**Figure 2:**
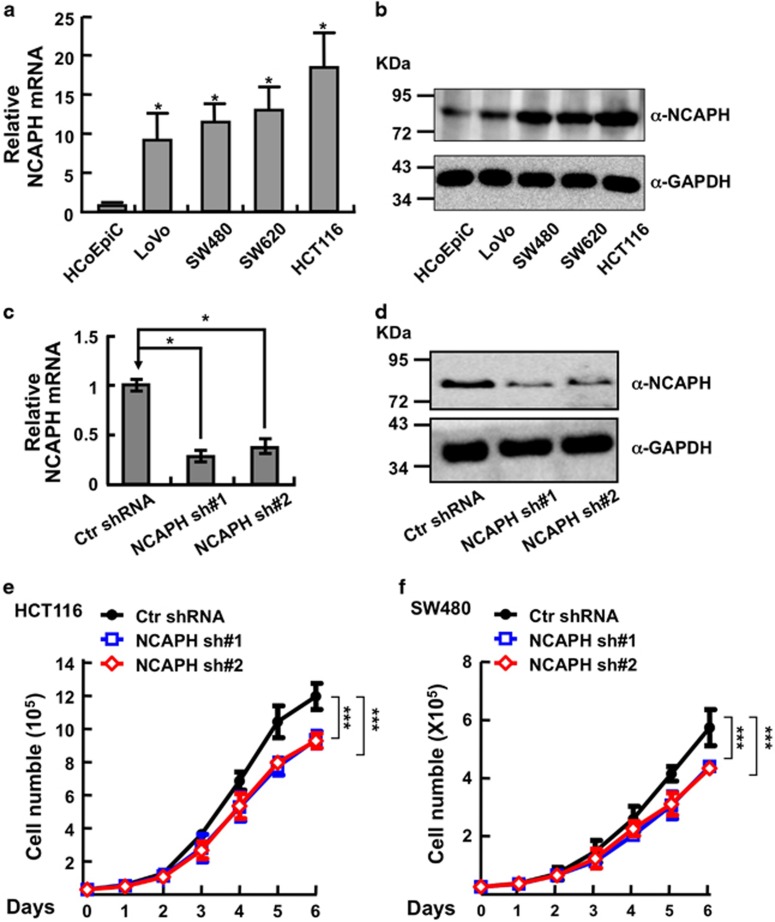
Knockdown of NCAPH inhibits colonic cancer cell proliferation. (**a**) Relative NCAPH mRNA expression in different colonic cell lines: normal human colonic epithelial cell (HCoEpiC) and colorectal cancerous cell lines (LoVo, SW480, SW620 and HCT116). HCoEpiC was set as a control. (**b**) Relative NCAPH protein expression in different colonic cell lines. Indicated cell lysates were probed with NCAPH and GAPDH antibodies, representative images were shown. (**c–-d**) Individual NCAPH-shRNA knockdown efficiency was verified by real-time PCR (**c**) and western blot with NCAPH and GAPDH antibodies (**d**); scramble shRNA was set as a control. *α*, anti; Ctr, scramble shRNA control; sh#1, shRNA#1; sh#2, shRNA#2. (**e** and **f**) Knockdown of NCAPH significantly inhibited HCT116 (**e**) and SW480 (**f**) cell growth. ***P<0.001, *t*-test

To examine the exact functional roles of NCAPH in colon cancers, we first analyzed the relative mRNA and protein expressions of NCAPH in normal human colonic epithelial cell and colorectal cancerous cell lines (LoVo, SW480, SW620 and HCT116). Consistent with our findings in colonic tissues, we found that both NCAPH mRNA and protein expressions are unanimously upregulated in all colorectal cancerous cell lines, with the highest expression in HCT116 (Figures 2a and b). Owing to the higher expression of NCAPH in colonic cancerous cell lines, we speculated that NCAPH might play important roles and be frequently mutated in human colon cancers. Consistent with our hypothesis, we identified many NCAPH mutations in colon cancers by applying the cBioPortal web resource^24^ (Supplementary Table 4), indicating that NCAPH is important for CC cell homeostasis.

### Depletion of NCAPH inhibits CC cell proliferation and xenograft tumor formation

**Figure 3:**
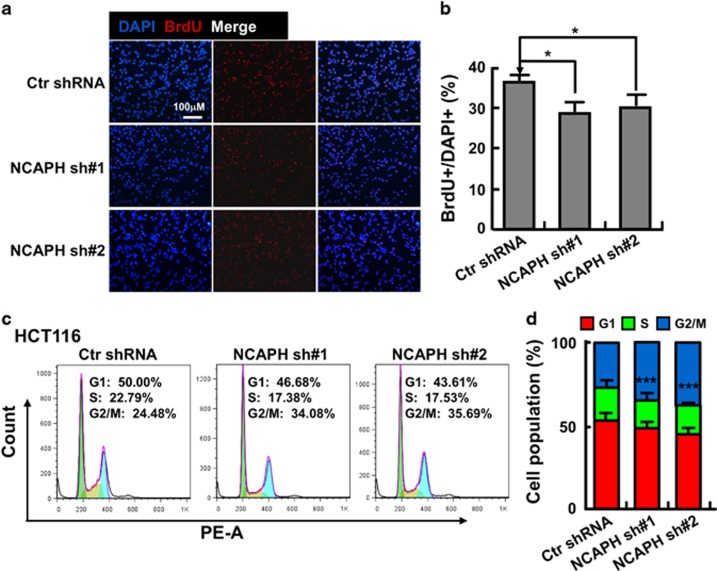
Knockdown of NCAPH inhibits cell proliferation and cell cycle transition. (**a**) NCAPH knockdown inhibited DNA synthesis in HCT116 cells by BrdU incorporation assay. (**b**) Quantification data for **a**. *P<0.05, **P<0.01, *t*-test. (**c**) Depletion of NCAPH results in accumulation of G2/M cells by FACS analysis. HCT116 cells stably expressing Ctr and NCAPH shRNAs individually were stained with PI for the analysis of cell cycle distribution. (**d**) Quantification data for **c**. ***P<0.001, *t*-test

**Figure 4:**
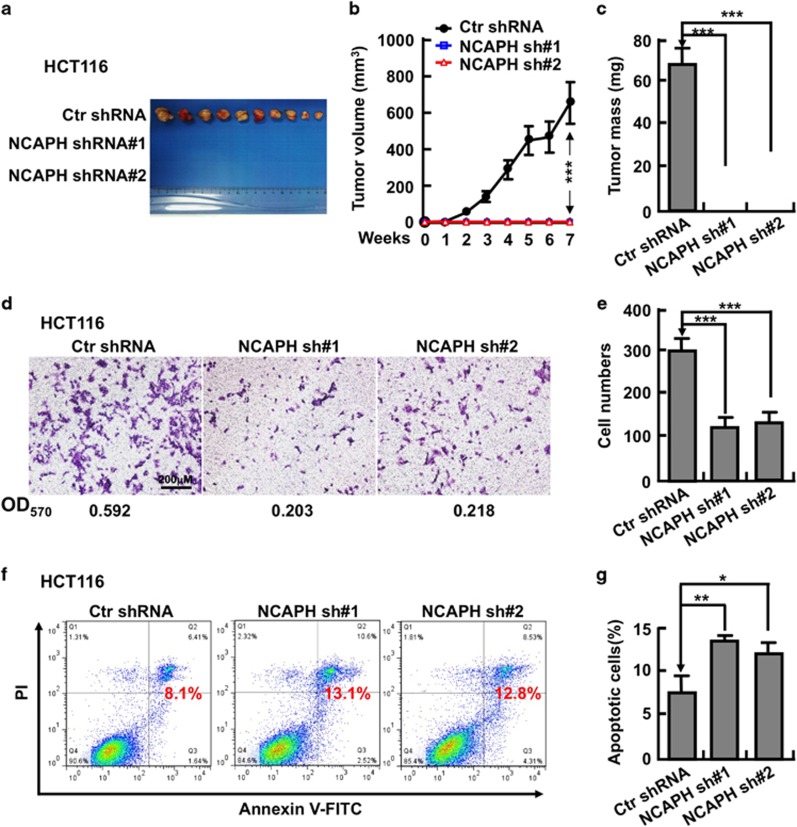
NCAPH knockdown promotes cell apoptosis, inhibits cell migration and xenograft tumor formation *in vivo*. (**a**) Tumor masses collected from HCT116 stably expressing Ctr or NCAPH shRNAs after tumors had grown for 7 weeks. (**b**) NCAPH knockdown significantly decreased xenograft tumor growth in male NOD SCID mice. ***P<0.001, *t*-test. (**c**) Depletion of NCAPH significantly suppressed xenograft tumor weights. ***P<0.001, *t*-test. (**d**) Knockdown of NCAPH by independent shRNAs decreased HCT116 transwell cell migration (48 hrs). The OD_570_ number was indicated below indicated images. (**e**) Quantification data for **d**. ***P<0.001, *t*-test. (**f**) Indicated cells were collected for annexin V staining and flow cytometry assays. (**g**) Quantification data for **f**. *P<0.05, **P<0.01, *t*-test

We then picked colonic cancerous cell lines HCT116 and SW480 for further studies. We inhibited the NCAPH mRNA expression with two independent lentiviral shRNAs targeting to different regions of NCAPH mRNA (Materials and Methods). We validated the shRNA knockdown efficiency by comparing both the relative mRNA and protein expression levels in NCAPH-shRNA targeted cell lines with that in scramble shRNA control cells (Figures 2c and d). As expected, stable knockdown of NCAPH inhibited HCT116 and SW480 cell proliferation *in vitro* (Figures 2e and f). In addition, NCAPH knockdown significantly decreased the ratio of BrdU-positive cells in HCT116 (Figures 3a and b). To verify whether NCAPH is important for colonic cancer cell growth *in vivo*, we performed a tumorigenesis assay in nude mice. A total of 15 male NOD SCID mice of 5 weeks of age were randomly divided into three groups that were injected with HCT116 cell lines stably expressing scramble control shRNA or two NCAPH-targeted shRNAs, respectively (1 × 10^6^ cells per point subcutaneously). Mice were monitored each day and tumor growth was measured every 3 days. As expected, we observed subcutaneous tumors in the scramble shRNA control group. However, no visible tumor was detected in NCAPH-targeted shRNA knockdown groups by the end of the experiment, suggesting that the colonic tumor growth was markedly retarded by NCAPH depletion compared with scramble shRNA-transformed cells. Furthermore, both tumor weights and volumes in the HCT116 depletion groups were markedly less than those of the HCT116-scramble shRNA group (Figures 4a–c).

### NCAPH regulates CC cell migration, cell cycle and apoptotic signaling pathways

We further tested whether NCAPH regulates CC cell migration. We performed transwell assay and found that inhibiting NCAPH expression in HCT116 indeed markedly decreased cell migration (Figures 4d and e). As condensins play pivotal roles in chromosome assembly and segregation during mitosis and meiosis,^6, 7^ and our result showed that knockdown of NCAPH inhibited HCT116 cell proliferation (Figures 2e and f), we hypothesized that NCAPH might be important for HCT116 cell cycle regulation. To test this hypothesis, we characterized the HCT116 cell cycle transition by flow cytometry analysis, and found that depletion of NCAPH increased the ratio of G2/M cell population (Figures 3c and d). To figure out whether cell cycle arrest leads to cell apoptosis by NCAPH knockdown, cells were collected for annexin V staining and flow cytometry assay. The percentages of apoptotic cells in NCAPH-targeted shRNAs cell lines were significantly higher than that in scramble shRNA control cells (Figures 4f and g). Our data indicated that NCAPH is important both for CC cell cycle transition and apoptosis.

To sum up, our data indicated that NCAPH might be a novel candidate biomarker for CC. However, further studies should be carried out using NCAPH alone or in combination with other CC-specific biomarker to develop new CC therapeutic strategies.

## Discussion

Condensin complexes are structural components of mitotic chromosomes and play an important role in driving chromosome condensation.^8, 25^ Former findings demonstrated that the homologs of *Xenopus* condensin subunits, barren-1/hCAP-H, hCAP-C, hCAP-E, CNAP1 and hCAP-G form a heteropentamer capable of inducing chromosome condensation in the *Xenopus* egg extract model.^26^ Human condensin complex I contain five subunits: two core subunits, CAP-E/SMC2 and CAP-C/SMC4, and other three non-SMC subunits, CAP-D2, CAP-G and CAP-H. The human homolog of XCAP-H/barren, named hCAP-H (human chromosome-associated protein H), and it is mapped to 2q11.2.^27^

Recently, Kimura *et al.*^28^ have proposed that CAP-D2 and CAP-G may participate in the activation of the CAP-E and CAP-C ATPases by a process directly involving their HEAT domains or by modifying the conformation of the SMC heterodimer. In *Drosophila*, the condensin subunit barren, the homolog of XCAP-H, is required for chromatid arm resolution at anaphase, which was attributed to barren protein association with topoisomerase II (TOPOII) and its modulation on TOPOII activity *in vitro*.^29^ However, in *Saccharomyces cerevisiae*, the barren homolog Brn1p is required for chromatid condensation but not TOPOII activity.^30^ Thus, these subunits may have more comprehensive functions in different organisms.

Studies demonstrated that NCAPH have essential role in condensin complex stability and resolution of sister chromatids. Some studies concluded that the condensin II subunit CAP–G2 plays an important function during erythropoiesis,^9^ myeloma^31^ and lung adenocarcinoma.^32^ Interestingly, another study demonstrated that both condensin I and II complexes exhibit an unexpected, marked estrogen-induced recruitment to estrogen receptor *α* active enhancers in interphase breast cancer cells.^33^ Amplification of *MYCN*, a member of the *MYC* proto-oncogene family, is frequently observed in neuroblastoma, and condensin complexes have also been reported to be effective anti-neuroblastoma targets.^34^ Others identified that hCAP-D3 can be a new biomarker for subtype-1 tumors that improves prognostication, and reveal androgen signaling as an important biologic feature of this potentially clinically favorable molecular subtype.^35^ However, studies about the correlation of NCAPH with human diseases such as cancer were barely reported.

In this study, we analyzed the functional roles of NCAPH in colon cancerous cell lines and examined the correlation of NCAPH expression with CC. For the first time, we identified that both NCAPH mRNA and protein expression levels are upregulated in colon cancerous cell lines and tissues. Depletion of NCAPH, inhibits colonic cancerous cell proliferation, migration, affects cell cycle transition and induces cellular apoptosis. More importantly, we found that knockdown of NCAPH decreased the xenograft tumor formation of HCT116 *in vivo*.

Although advances in treatment, such as combination chemotherapy and chemoradiation, have slightly improved the outcome of tumor therapy over the last several decades, tumors are the leading cause of death in economically developed and developing countries.^36^ Colorectal tumors, lung tumors and multiple myeloma (a hematopoietic tumor) are particularly intractable. Therefore, novel drugs having potent activity are required to treat such tumors, and to develop them, it is important to identify novel molecular targets related to the pathogenesis of these tumors. Our study showed that NCAPH was expressed much higher in the CC tissues compared with adjacent normal tissues, which was statistically significant. And, we also found that the NCAPH high expression in tumor tissues of colon patients had a significantly better prognosis and survival rate than low-expression patients. However, the NCAPH high expression in adjacent normal tissues of colon patients showed no significant prognosis and survival rate compared with low-expression patients. It is implied that NCAPH high expression in the cytoplasm of tumor tissues, not in the cytoplasm of adjacent normal tissues is very significant and important for prognosis. Thus, NCAPH have an essential role in CC prognosis and treatment, and the results are consistent with a role of NCAPH in mitosis and chromosome condensation.

There are many key regulators or signaling pathways that play important roles in colorectal cancer chemo- and radio-therapies, such as p53, NF*κ*B, non-coding RNAs and etc.^37, 38, 39^ At this time, there is a lack of gene expression data derived from CC patients that could suggest that NCAPH high level expression is important for predicting sensitivity to chemo- and/or radio-therapies. However, our results indicate that patients with NCAPH high expression in colon tumor tissues might be associated with better prognosis, which could be explained by NCAPH sensitizing highly proliferating colon cancerous cells responding to chemo- and/or radio-therapies. To uncover the detail mechanisms, the exact interaction and functional complex of NCAPH, gene expression data and signaling pathways affected by NCAPH high expression need to be further characterized. We identified NCAPH high expression in colon tumor tissues as a biomarker that has been found in a majority of CC. Given the exploratory and retrospective design of our study, these results will need to be further validated. We advocate for these findings to be confirmed within the framework of randomized, clinical trials, in conjunction with genomic DNA sequencing studies. Taken together, we also provide new insight the prognosis and diagnosis treatment of CC.

## Materials and Methods

### Constructs and cell culture

Independent shRNAs targeting to NCAPH were constructed using pLKO.1 vector, constructs were sequence verified and 21 bp targeting sequences are: shRNA#1, 5′-TCAGAGATTCTTAAACAGAAA-3′ shRNA#2, 5′-TCTCCTAAATTGATCTGTTAT-3′. The control scramble shRNA sequence is: 5′-GCACTACCAGAGCTAACTCAG-3′. Lenti-viruses were generated according to the manufacture protocol, cells were infected by viruses twice with 48 and 72 h viral supernatants containing 4 *μ*g ml^−1^ polybrene. HEK-293T cells were obtained from ATCC (Manassas, VA, USA) and cultured following standard protocol. Colonic cell lines were cultured in RPMI-1640 medium supplemented with 10% fetal bovine serum and 1% penicillin/streptomycin, and then were incubated in a humidified atmosphere with 5% CO_2_ at 37 °C.

### Cell proliferation, migration assays and xenograft tumor model

Indicated cells were plated into 24-well plates, the cell numbers were subsequently counted each day. The migration ability of indicated cells were evaluated by transwell assay using 24-well chemotaxis chambers (Corning cell culture inserts (Corning, NY, USA), 8 *μ*m pore size). For xenograft tumor growth experiment, total 15 male NOD SCID mice at 5 weeks of age were divided into indicated groups and injected with indicated cell lines, each experiment were repeated at least three times. Tumor sizes in all groups were measured every 3 days for 7 weeks using Vernier calipers (Suzhou, China). All mice were killed at the end of the experiment and tumors were collected and weighed.

### Hematoxylin and eosin staining

The colon TMAs contain 90 pairs of CC tumors together with matched adjacent normal colon tissues, which were collected taking necessary precautions. The paraffin-embedded sections were prepared and used for histological and immunohistochemical analysis. The slices were cut into 5 *μ*m sections and stained with hematoxylin–eosin for histological evaluation and determined by analysis under a microscope (Olympus, Tokyo, Japan).

### Immunohistochemical

The sections were placed in an incubator for 2 h at 80 °C and then deparaffinized in xylene, rehydrated through graded ethanol solutions and immersed in a 0.3% hydrogen peroxide solution in methanol for 15 min to inhibit endogenous peroxidase activity. After washing with Tris buffer saline (TBS), antigen retrieval was performed by placing the slides at 95 °C for 20 min in a microwave oven and allowed to cool for 1 h at room temperature. The slides were again washed three times with TBS, and nonspecific binding was blocked by pre-incubation with 5% goat serum for 30 min at room temperature. Slide was then incubated for 2 h at 4 °C with primary anti-NCAPH antibody (Proteintech, 11515-1-AP, Chicago, IL, USA) at a dilution of 1 : 200 in the blocking buffer. After washing the slides three times with TBS, sections were subsequently treated with HRP-labeled second antibody (Santa Cruz Biotechnology, Santa cruz, CA, USA) for 30 min. Diaminobenzidine was used as a chromogen followed by slight hematoxylin counterstaining. The slides were then dehydrated, cleared with xylene and mounted with dibutylphthalate xylene. The slides were determined by analysis under a microscope (Olympus).

### Analysis of TMAs

Colon cancer TMAs, fully annotated with clinical and pathological information, were obtained from Shanghai outdo Biotech Co., Ltd (Shanghai, China) (catalog no. HRec-Ade180Sur-05). A detailed description of the patient represented in each TMA and of the scoring system used to evaluate NCAPH expression is provided. A staining score was equal to the dyeing intensity score and the dyeing positive cells ratio. The dyeing intensity with scores of 0 (negative), 1 (weak), 2 (intermediate), 3 (strong); the dyeing positive cells ratio with scores of 0 (negative), 1 (1–25%), 2 (26–50%), 3 (51–75%) and 4 (76–100%). The staining score <8 as low antibody expression groups, and ≥8 as high antibody expression groups.

### Statistical analysis

Patient subgroups were compared with respect to survival outcomes with the use of Kaplan–Meier curves, log-rank tests and multivariate analyses based on the Cox proportional hazards method. Each experiment was performed at least three times. Differences were considered significant if the *P*-value was <0.05 (*P<0.05, **P<0.01, ***P<0.001), compared with the control group.
