# Supplementary material for: NCAPH plays important roles in human colon cancer
Source: Cell Death Dis. 2017 Mar 16;8(3):e2680–. doi: 10.1038/cddis.2017.88 (PMC5386579; doi:10.1038/cddis.2017.88)
Supplement: Supplementary Information [file cddis201788x1.doc]

**Table 1. Clinical and pathological features of 90** patients with colon cancer.

| Variables | | Total n=90 |
| --- | --- | --- |
| Tumor grade | Ⅰ,Ⅱ | 48 |
|  | Ⅲ | 42 |
| TNM staging | stage-1 | 8 |
|  | stage-2 | 48 |
|  | stage-3 | 30 |
|  | stage-4 | 2 |
| Gender | Male | 47 |
|  | Female | 42 |
| Age | ≤65 | 32 |
|  | ＞65 | 53 |
| Tumor size | ≤5cm | 45 |
|  | ＞5cm | 44 |

**Table 2. Survival distributions for the different levels of NCAPH expression in colon tumor tissues.**

| **Case Processing Summary** | | | | |
| --- | --- | --- | --- | --- |
| **NCAPH expression** | **Total N** | **N of Events** | **Censored** | |
|  |  |  | **N** | **Percent** |
| Low | 12 | 11 | 1 | 8.3% |
| High | 75 | 41 | 34 | 45.3% |
| Overall | 87 | 52 | 35 | 40.2% |

| **Overall Comparisons** | | | |
| --- | --- | --- | --- |
|  | Chi-Square | df | Sig. |
| Log Rank (Mantel-Cox) | 7.203 | 1 | 0.007 |

**Table 3. Survival distributions for the different levels of NCAPH expression in adjacent normal tissues.**

| **Case Processing Summary** | | | | |
| --- | --- | --- | --- | --- |
| NCAPH expression | Total N | N of Events | Censored | |
|  |  |  | N | Percent |
| Low | 63 | 37 | 26 | 41.3% |
| high | 24 | 16 | 8 | 33.3% |
| Overall | 87 | 53 | 34 | 39.1% |

| **Overall Comparisons** | | | |
| --- | --- | --- | --- |
|  | Chi-Square | df | Sig. |
| Log Rank (Mantel-Cox) | .893 | 1 | .345 |

**Table 4. NCAPH mutation information in human colon cancers**

| **Sample ID** | **Cancer Study** | **AA change** | **Type** |
| --- | --- | --- | --- |
| coadread_dfci_2016_255 | Colorectal (DFCI 2016) | G21R | Missense |
| coadread_dfci_2016_2955 | Colorectal (DFCI 2016) | P37L | Missense |
| coadread_dfci_2016_3704 | Colorectal (DFCI 2016) | L736R | Missense |
| coadread_dfci_2016_2269 | Colorectal (DFCI 2016) | D731N | Missense |
| coadread_dfci_2016_1849 | Colorectal (DFCI 2016) | L409I | Missense |
| coadread_dfci_2016_306558 | Colorectal (DFCI 2016) | T222N | Missense |
| coadread_dfci_2016_2227 | Colorectal (DFCI 2016) | D469Ifs*25 | FS del |
| coadread_dfci_2016_1241 | Colorectal (DFCI 2016) | D469Ifs*25 | FS del |
| coadread_dfci_2016_2765 | Colorectal (DFCI 2016) | E234K | Missense |
| coadread_dfci_2016_3658 | Colorectal (DFCI 2016) | V197A | Missense |
| 587238 | Colorectal (Genentech) | G321R | Missense |
| 587222 | Colorectal (Genentech) | E147D | Missense |
| 587304 | Colorectal (Genentech) | D612H | Missense |
| 587350 | Colorectal (Genentech) | R696M | Missense |
| TCGA-AA-A00N-01 | Colorectal (TCGA pub) | D54A | Missense |
| TCGA-AA-A00E-01 | Colorectal (TCGA pub) | L658F | Missense |
| TCGA-AA-A01Q-01 | Colorectal (TCGA pub) | I315T | Missense |
| TCGA-AA-3977-01 | Colorectal (TCGA pub) | D366N | Missense |
| TCGA-AA-A010-01 | Colorectal (TCGA pub) | I633T | Missense |
| TCGA-AA-A01Q-01 | Colorectal (TCGA) | I315T | Missense |
| TCGA-AA-A00E-01 | Colorectal (TCGA) | L658F | Missense |
| TCGA-AA-3977-01 | Colorectal (TCGA) | D366N | Missense |
| TCGA-AA-A00N-01 | Colorectal (TCGA) | D54A | Missense |
